# Case Report: Prenatal Identification of a *De Novo* Mosaic Neocentric Marker Resulting in 13q31.1→qter Tetrasomy in a Mildly Affected Girl

**DOI:** 10.3389/fgene.2022.906077

**Published:** 2022-07-19

**Authors:** Avinash V. Dharmadhikari, Elaine M. Pereira, Carli C . Andrews, Michael Macera, Nina Harkavy, Ronald Wapner, Vaidehi Jobanputra, Brynn Levy, Mythily Ganapathi, Jun Liao

**Affiliations:** ^1^ Department of Pathology & Cell Biology, Columbia University Vagelos College of Physicians and Surgeons and New York-Presbyterian Morgan Stanley Children’s Hospital, New York, NY, United States; ^2^ Department of Pediatrics, Columbia University Vagelos College of Physicians and Surgeons and New York-Presbyterian Morgan Stanley Children’s Hospital, New York, NY, United States; ^3^ Department of Pediatrics, Columbia University Vagelos College of Physicians and Surgeons, New York, NY, United States; ^4^ Clinical Cytogenetics Laboratory, New York Presbyterian Morgan Stanley Children’s Hospital, New York, NY, United States; ^5^ Department of Obstetrics and Gynecology, Columbia University Vagelos College of Physicians and Surgeons and New York-Presbyterian Morgan Stanley Children’s Hospital, New York, NY, United States

**Keywords:** supernumerary marker chromosome, chromosomal microarray, non-invasive prenatal screening, 13q31.1, neocentromere

## Abstract

Partial tetrasomy of distal 13q has a reported association with a variable phenotype including microphthalmia, ear abnormalities, hypotelorism, facial dysmorphisms, urogenital defects, pigmentation and skin defects, and severe learning difficulties. A wide range of mosaicism has been reported, which may, to some extent, account for the variable spectrum of observed phenotypes. We report here a pregnancy conceived using intrauterine insemination in a 32-year-old female with a history of infertility. Non-invasive prenatal screening (NIPS) was performed in the first trimester which reported an increased risk for trisomy 13. Follow-up cytogenetic workup using chorionic villus sampling (CVS) and amniotic fluid samples showed a mosaic karyotype with a small supernumerary marker chromosome (sSMC). Chromosomal microarray analysis (CMA) identified a mosaic 31.34 Mb terminal gain on chr13q31.1q34 showing the likely origin of the sSMC to distal chromosome 13q. Follow-up metaphase FISH testing suggested an inverted duplication rearrangement involving 13q31q34 in the marker chromosome and the presence of a neocentromere. At 21 months of age, the proband has a history of gross motor delay, hypotonia, left microphthalmia, strabismus, congenital anomaly of the right optic nerve, hemangiomas, and a tethered spinal cord. Postnatal chromosome analyses in buccal, peripheral blood, and spinal cord ligament tissues were consistent with the previous amniocentesis and CVS findings, and the degree of mosaicism varied from 25 to 80%. It is often challenging to pinpoint the chromosomal identity of sSMCs using banding cytogenetics. A combination of low-pass genome sequencing of cell-free DNA, chromosomal microarray, and FISH enabled the identification of the precise chromosomal rearrangement in this patient. This study adds to the growing list of clinically identified neocentric marker chromosomes and is the first described instance of partial tetrasomy 13q31q34 identified in a mosaic state prenatally. Since NIPS is now being routinely performed along with invasive testing for advanced maternal age, an increased prenatal detection rate for mosaic sSMCs in otherwise normal pregnancies is expected. Future studies investigating how neocentromeres mediate gene expression changes could help identify potential epigenetic targets as treatment options to rescue or reverse the phenotypes seen in patients with congenital neocentromeres.

## Introduction

Neocentromeres are new centromeres formed in chromosomal locations other than the original centromere. Despite the complete absence of normal alpha-satellite DNA, a tandem repeat sequence featuring in all eukaryotic endogenous centromeres, neocentromeres can bind all known essential centromere proteins, assemble a functional kinetochore, and behave normally in mitosis and meiosis. Current experimental evidence indicates that neocentromere activity is acquired epigenetically rather than by altering the DNA sequence. Also, there is no apparent sequence specificity for neocentromere-containing regions. However, specific sequence preferences for neocentromere formation have been reported, including high AT contents (Lo et al., 2001a; Lo et al., 2001b; Alonso et al., 2003; and Murmann et al., 2009), and the presence of long interspersed nuclear element (LINE) repeats (Chueh et al., 2005; Alonso et al., 2007; Murmann et al., 2009).

Since the initial discovery in 1993 (Voullaire et al., 1993), over 100 cases of constitutional neocentromere formation in humans have been reported in the literature (Marshall et al., 2008; Naughton and Gilbert, 2020). The majority of them are small supernumerary marker chromosomes (sSMCs), resulting from inverted duplications of the distal segments of a chromosome arm. Neocentromeric sSMCs are often present in the individual in a mosaic form, probably due to mitotic instability or selective disadvantage. The precise mechanism for the formation of inverted duplicated sSMCs is currently unknown. However, due to the genotype similarity between the arms of neocentromeric sSMCs, it has been proposed that they are derived after a double-strand break and an intra-chromosomal exchange. At the same time, a neocentromere is formed (Murmann et al., 2009).

Inverted duplicated sSMCs with neocentromeres are commonly present in addition to two normal chromosomes. In rare cases, one normal chromosome and one derivative chromosome with the same region deleted can be present, thus resulting in tetrasomy or trisomy for the terminal chromosomal region present on the sSMC. Clinically, they are associated with developmental delay, intellectual disability, and/or congenital abnormalities in most reported cases due to large unbalanced genomic regions. For this reason, they also provide us with a valuable opportunity to evaluate the clinical consequences of dosage changes in large genomic regions without interferences from any other chromosomes, unlike cases with unbalanced translocations (Tharapel et al., 1986).

Chromosome 13q contains several “hotspots” for neocentromere formation, including 13q32, 13q31, and 13q21 (Warburton et al., 2000; Li et al., 2002). Approximately 20 patients with inverted duplicated 13q have been reported in the medical literature (Marshall et al., 2008; Mascarenhas et al., 2008; Haddad et al., 2012; Yu et al., 2012; Stembalska et al., 2015). Genotype–phenotype correlation analyses suggest that partial tetrasomy of distal 13q is associated with a variable phenotype including microphthalmia, ear abnormalities, hypotelorism, facial dysmorphisms, urogenital defects, pigmentation and skin defects, and severe learning difficulties (Barwell et al., 2004; Dhar et al., 2009; Myers et al., 2015). However, those analyses are often limited due to the conventional banding cytogenetic methods used and the mosaic nature of the sSMCs. Here, we report a prenatally identified mosaic sSMC with a neocentromere and the resulting tetrasomy of distal chromosome 13q detected using a combination of diverse techniques, including low-pass genome sequencing of cell-free DNA, high-resolution SNP cytogenomic microarray, FISH, and conventional banding cytogenetics.

## Case Description

Our patient is the child of a 36-week gestation to a 32-year-old mother of German/Italian descent and a father of Irish descent. The patient’s conception resulted from intrauterine insemination. Non-invasive prenatal screening (NIPS) performed in the first trimester reported an increased risk for trisomy 13. At 27-week gestation, fetal ultrasound and echocardiogram were normal. Though oligohydramnios was noted at 33 weeks, the remainder of the pregnancy and delivery was uncomplicated. After birth, the patient required nasal CPAP for respiratory distress and was eventually transferred to the neonatal intensive care unit (NICU) for further management. A physical examination shortly after birth was remarkable for slight posterior rotation of the left ear, uplifted ear lobes, bulbous nasal tip, arched palate, slight neck skin redundancy, and bilateral fifth finger and toe clinodactyly. Her respiratory distress resolved within a day. A head ultrasound was performed within the first day of life showed nonspecific thinning of the corpus callosum. She was discharged on day of life 3.

A three-generation pedigree was completed during the initial evaluation. Maternal family history is significant for an older half-sister with a history of partial seizures from age 3 to 6 years and an uncle with a history of hemangiomas in infancy. Paternal family history is significant for an aunt with an unspecified congenital heart condition and two male cousins with a severe autism spectrum disorder.

**FIGURE 1:**
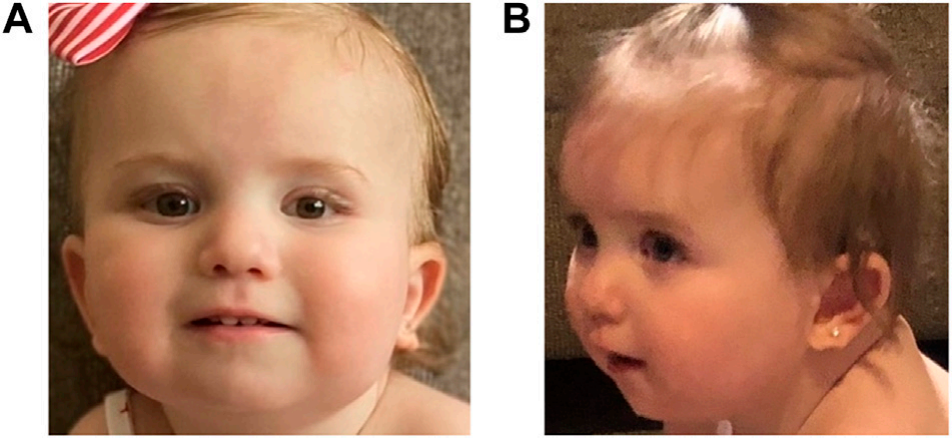
Pictures of the proband showing mild dysmorphic features. In both pictures, the proband is slightly tilting her head up; panel **(A)**: an epicanthal fold is noted on the right. On the left, mild microphthalmos is noted. Her ears are standard set with posterior rotation; panel **(B)**: her left ear lobe is notched. The proband has a thin upper lip.

The patient established care with multiple subspecialty providers. At 2 months old, she was evaluated by a neurologist for concerns of abnormal hand movements. An EEG at 4 months of age was normal. Hypotonia was noted at 4 months, and an early intervention evaluation was recommended; she obtained speech and physical therapy. Ophthalmology evaluations showed an abnormal right optic nerve and left microphthalmos. At 5 months of age, she established care with dermatology for red pigmentation over her face, neck, buttocks, back, and genitalia, concerning for capillary malformations. At 7 months of age, she was referred to neurosurgery for a skin lesion in the sacral area, concerning for a spinal dysraphism. Workup, including a spinal MRI, showed evidence of a tethered cord. The patient underwent corrective surgery at 1 year old. At 14 months of age, the patient began to have feeding difficulties. A modified barium swallow showed aspiration. The patient started crawling at 16 months and was standing and cruising at 17 months old. She still exhibited speech delay at 17 months of age as she babbled but was not yet articulating words. She was able to feed herself and indicate her wants. The patient was also able to dress and undress with her parents’ assistance. At 21 months of age, she began to use a G-tube for feeding to prevent aspiration. Cardiac and audiology evaluations have been normal to date. Following her most recent early intervention evaluation, she qualified for feeding, speech, physical, occupational therapies, and special instruction (Figures 1A,B).

As a follow-up to the NIPS result, confirmatory genetic testing on chorionic villus sampling (CVS) tissue was performed at a commercial laboratory. Subsequently, diagnostic genetic testing on amniotic fluid and postnatal tissues was performed at our center in the Clinical Cytogenetics Laboratory and the Laboratory of Personalized Genomic Medicine at the Columbia University Medical Center, New York, NY. Cytogenetic analysis on amniotic fluid, peripheral blood, and buccal and ligament tissues was carried out according to the standard methods (Arsham et al., 2017). Chromosomal microarray (CMA) was performed on DNA isolated from an amniotic fluid sample with the CytoScan^®^ HD platform (Affymetrix) as previously outlined (Ganapathi et al., 2019). A whole-exome sequencing (WES) for the proband and her parents was performed on DNA extracted from peripheral blood mononuclear cells as previously described (Abdelhakim et al., 2020). Written consent was obtained from the minor’s legal guardians for genetic studies and inclusion, in this case study of any potentially identifiable images or data as per hospital protocol.

**FIGURE 2:**
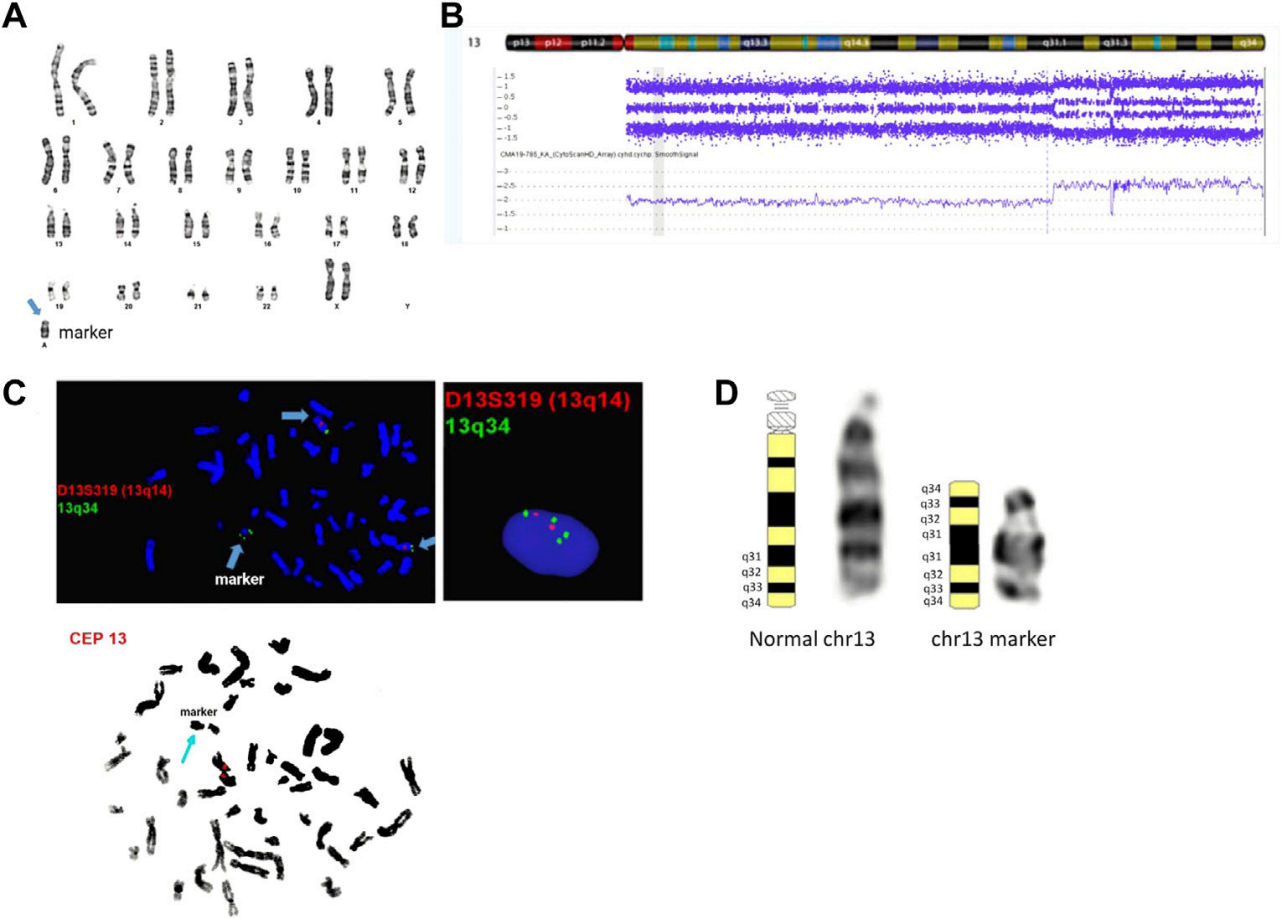
Cytogenetic and genomic characterization of the sSMC. Panel **(A)**: female karyotype with a small supernumerary marker chromosome identified by G-banding from amniotic fluid; banding resolution: 400-band-level; panel **(B)**: SNP-based microarray results showing 31.34 Mb gain in the distal end of chromosome 13q: arr [GRCh37] 13q31.1q34 (83,666,539_115,010,330)x2∼4; probe targets: 2,696,550; human genome build (hg19); panel **(C)**: metaphase and interphase FISH results using probes mapping to 13q14 (D13S319), 13q34 (LAMP1), and chr 13 centromere. The final karyotype is: 47,XX,+mar[28]/46,XX[2]. nuc ish (D13S319x2,LAMP1x4)[122/300]. ish der (13) (D13S319,LAMP1++) (CEP13-); panel **(D)**: chromosome 13 ideograms showing representative normal and marker chromosome 13 in the patient. The long form description of the marker chromosome cell line as per ISCN 2020 is as follows: 47,XX,+dup (13) (qter- > q31.1::q31.1- > q33- > neo- > q33- > qter).

Cytogenetic analysis from an amniotic fluid sample showed a mosaic karyotype with a small supernumerary marker chromosome (sSMC) (Figure 2A). CMA identified a mosaic 31.34 Mb terminal gain: arr [GRCh37] 13q31.1q34 (83,666,539_115,010,330) x2∼4. No other pathogenic, likely pathogenic, or uncertain clinically significant CNVs were detected by CMA. SNP probes from the microarray analysis showed four genotypes in the 13q gain, including either 0:4 (AAAA and BBBB) or 1:3 (AAAB and ABBB) genotype ratios for A and B alleles and the absence of 2:2 genotype ratio (AABB) (Figure 2B), which indicates that SNPs on both arms of the duplicated sSMC are identical and they are originated from the same chromosome fragment. Follow-up interphase and metaphase FISH testing using probes specifically for 13q14 and 13q34 showed the level of mosaicism of the chromosome 13-derived sSMC to be approximately 40% and suggested an inverted duplication rearrangement involving 13q31q34, consistent with the G-banding pattern seen on the sSMC. Additionally, metaphase FISH testing using the centromeric CEP13 probe failed to hybridize on the sSMC suggesting the presence of a neocentromere (Figure 2C). The marker chromosome was submetacentric in appearance with a primary constriction suggesting the presence of the neocentromere most likely at chromosomal band 13q33 (Figure 2D). The final karyotype is: 47,XX,+mar[28]/46,XX[2]. nuc ish (D13S319x2,LAMP1x4)[122/300]. ish der (13) (D13S319,LAMP1++) (CEP13-) (the long-form description of the marker chromosome cell line as per ISCN 2020 is as follows: 47,XX,+dup (13) (qter- > q31.1::q31.1- > q33- > neo- > q33- > qter)). Parental karyotypes were normal, suggesting *de novo* origin of the marker chromosome.

Postnatal chromosome and FISH analyses in buccal, peripheral blood, and spinal ligament (post-laminectomy) tissues were consistent with the previous amniocentesis and CVS findings. The degree of mosaicism varied from 25 to 80% (Table 1). Given the capillary malformations, whole-exome sequencing (WES) was requested and was negative (no pathogenic/likely pathogenic variants or variants of uncertain significance VUS(s) related to the patient’s phenotype), except for a maternally inherited pathogenic variant in the *BRCA2* gene. Given the identification of a maternally inherited pathogenic *BRCA2* variant, familial cascade testing has been initiated.

**TABLE 1:** Degree of mosaicism of the sSMC in different tissues.

Tissue	% mosaicism (FISH) (# cells)
Amniotic fluid	41 (122/300)
Buccal swab	25 (106/430)
Peripheral blood	50 (199/400)
Spinal ligament (from laminectomy)	80 (403/500)

The region of copy number gain in the patient in this report was 31.34 Mb in size; genomic coordinates: chr13:83,666,539–115,010,330 (hg19) encompassing 195 genes, of which 83 genes are OMIM-annotated. OMIM disease genes include *COL4A1*, *COL4A2*, *ZIC2*, and *SLITRK1*, associated with autosomal dominant (AD) disorders; *CARS2*, *CLDN10*, *DNAJC3*, *DCT*, *ERCC5*, *FGF14*, *F7*, *F10*, *GPC6*, *GRK1*, *LIG4*, *NAXD*, *PCCA*, *SLC10A2*, *SLITRK6*, *TGDS*,and *TPP2* associated with autosomal recessive (AR) disorders; and *DZIP1* and *NALCN* associated with both AD/AR conditions.

## Discussion

The American College of Obstetrics and Gynecology (ACOG) recommends non-invasive prenatal screening for all women, regardless of maternal age (American College of Obstetricians and Gynecologists’ Committee on Practice Bulletins—Obstetrics, 2020). In addition to detecting trisomies for chromosomes 21, 18, and 13 and sex chromosome aneuploidies, expanded NIPS tests based on a whole-genome assessment of cell-free DNA in maternal plasma are able to identify rare autosomal trisomies (RATs), known microdeletions/duplications, and rare copy number and structural variants (Chitty, 2021). Recent reports in the literature describe and discuss the detection of marker chromosomes by NIPS (Luo et al., 2020; Liehr, 2021). They are initially detected as copy number gains and require confirmatory testing by karyotype, FISH, and/or CMA to determine their exact genomic content. The mosaic nature of these sSMCs makes detection and prediction of clinical consequences often a diagnostic and clinical challenge. We summarize findings in a patient where NIPS detected a copy number gain in chromosome 13, raising concern for trisomy 13. Confirmatory testing using conventional cytogenetic techniques and SNP-based chromosomal microarray analysis helped decipher the precise chromosomal location and orientation of this additional genomic content to chromosome 13q31.1q34.

While chromosome 13q is a known hotspot for neocentric sSMCs with multiple case reports described in the literature, the marker’s characterization of the genomic content is often limited to karyotype and FISH. There are seven previously published cases concerning patient reports involving markers with breakpoints mapping to 13q31 (Table 2, Supplementary Table S1) (Tohma et al., 1998). Overlapping features in four mosaic cases identified postnatally include growth delay, hypertelorism, strabismus, abnormal ears, extra teeth, mild psychomotor delays, and seizures (Li et al., 2002; Yu et al., 2012; Myers et al., 2015). The degree of mosaicism in these patients ranged from 13 to 60%. Prenatal reports are few and include two non-mosaic cases with severe cystic hygroma, short and long bones, cerebellar hypoplasia, renal dysplasia, and early terminations (Mascarenhas et al., 2008; Haddad et al., 2012). There is also an additional report of hexasomy due to two 13q31q34 marker chromosomes in a fetus with increased nuchal translucency and similar prenatal findings (Stembalska et al., 2015). The clinical presentation in the current patient consists of mildly dysmorphic ears, clinodactyly, hypotonia, speech delay, nonspecific thinning of the corpus callosum, microphthalmia, concerning for capillary malformations, and a tethered spinal cord requiring laminectomy.

**TABLE 2:** Summary of chromosomal findings, degree of mosaicism, inheritance, and clinical symptoms in the current proband and previously reported sSMC cases with breakpoints in 13q31.

Author	Chromosomal finding	Mosaicism (%)	Inheritance	Clinical symptom
Current study	47,XX,+mar[28]/46,XX[2].nuc ish (D13S319x2,LAMP1x4)[122/300].ish der (13) (D13S319,LAMP1++), (CEP13-); arr [GRCh37] 13q31.1q34 (83,666,539_115,010,330)x2∼4 (31.34 Mb)	Amniotic fluid (41%), buccal (25%), peripheral blood (50%), and spinal ligament (80%)	*De novo*	Gross motor delay, hypotonia, left microphthalmia, oculomotor apraxia, strabismus, mild dysmorphism, congenital anomaly of the right optic nerve, hemangiomas, and a tethered spinal cord requiring laminectomy
Tohma et al. (1998)	47,XY,+inv dup (13) (qter- > q31::q31neo- > qter)	Blood (60%)	Unknown	Scoliosis, intestinal malrotation, hypospadias, hydronephrosis, mild dysmorphic features, strabismus, learning difficulties, seizures, patent ductus arteriosus, diaphragmatic hernia, bronchial anomalies, and extra teeth
Li et al.( 2002)	47,XX,+ inv dup (13) (qter- > q31::q31- > q32 neo- > qter)	Blood (54%)	*De novo*	Mild dysmorphic features, clinodactyly, strabismus, mild developmental delays, extra low incisor, and nevus flammeus on the nasal bridge
Barwell et al. (2004)	47,XX,+der (13) (qter- > q31neo::q31 - > qter)[8] *de novo*/46,XX [52].ish der (13) (wcp13+,13/21cen-,D13S585+ +,D13S1825++)	Blood and skin fibroblasts (13%)	*De novo*	Mild motor developmental delay, learning difficulties, seizures, extra teeth, unilateral hearing loss, soft dysmorphic features, arm hemihypertrophy, torticollis, and head circumference on the 98th percentile
Yu et al. (2012)	47,XY,+inv dup (13)[8]/46, XY[12].ish inv dup (13) (p-acro–,D13Z1/D21Z1–,WCP13+)arr [GRCh36] 13q31.3qterx2∼3 (20.77 Mb); additional findings of arr [GRCh36] 15q13.3x3 (495 kb, *de novo*), 16p12.1x1 (580 kb, mat), 16p11.2x3 (410 kb, mat)	Blood (40%)	*De novo*	Learning difficulties, cleft palate, and seizures
Mascarenhas et al. (2008)	47,XX,+mar.ish inv dup (13)	Non-mosaic	*De novo*	Oligohydramnios, large cisterna magna, ventriculomegaly, enlarged and hyperechogenic kidneys, club left foot, thymic hypoplasia, mild dysmorphic features, and pregnancy terminated
	(qter- > q31::q31- > neo- > qter) (wcp13+			
	D13Z1/D21Z1–,D13S327++)			
Haddad et al. (2012)	47,XY,+mar.ish inv dup (13) (qter→q31.1::q31.1→ qter) (wcp13+, YAC 921F2-	Non-mosaic	*De novo*	Dysmorphic features , cystic cervical hygroma, postaxial polydactyl of the right hand and left foot with short fingers, malrotation of the gut, micropenis with hypospadias, and pregnancy terminated
	D13Z1/D21Z1-, YAC 935C1++, RP11-569D9++).arr 13q31			
	1q34 (81,994,976_114,871,440)x4 [100%] (32.9 Mb)			
Stembalska et al. (2015)	48,XX,+mar1,+mar2 inv	Non-mosaic	*De novo*	Increased nuchal translucency, dysmorphic facial features, head and body disproportion, ambiguous genitalia, shortening of long bones, incorrect positing of anus, and pregnancy terminated
	dup (13) (qter- > q31.3::q31.3- > qter)			
	arr [GRCh37] 13q31.3q34 (92507936_115092648)x5 (22.6 Mb)			

Note: Refer to the following database for previous cases: http://cs-tl.de/DB/CA/sSMC/0-Start.html.

The phenotypes seen in mosaic patients with distal 13q31 tetrasomy are milder than those seen in trisomy 13 patients who were presented with a severe phenotype with defects of the eye, nose, lip, holoprosencephaly, polydactyly, and skin defects. One reason for the milder phenotype is the mosaic occurrence of these markers. However, the degree of mosaicism was as high as 80% in spinal cord tissue of the patient in this study. It could suggest that a critical region responsible for the severity of trisomy 13 may exclude the distal portion of chromosome 13q31 and beyond. Similarly, based on a milder phenotype in a patient with triplication of 13q31.1q34, it was proposed that the critical region for trisomy 13 syndrome lies closer to the centromere in the 13q14-13q32.1 chromosomal interval (Krygier et al., 2014). The lack of association of holoprosencephaly in patients with duplications involving *ZIC2* in 13q32.3 (Jobanputra et al., 2012), and a mild phenotype consisting of postaxial polydactyly in a patient with an interstitial duplication of 13q31.1q32.1 (van der Zwaag et al., 2010), likely supports this proposed critical region.

Previous studies have suggested multiple candidate genes for the different phenotypes seen in patients with tetrasomy 13q. *GPC5* and *GPC6* have been proposed as candidate genes for the reported polydactyly (van der Zwaag et al., 2010) based on their putative role as cell surface heparan sulfate proteoglycans in the control of cell growth and cell division (Veugelers et al., 1999). While bi-allelic pathogenic variants in *GPC6* are causative of an autosomal recessive skeletal dysplasia syndrome omodysplasia 1 (MIM#258315), *GPC5* is not associated with a genetic disorder to date. However, the absence of polydactyly in the current patient contradicts the proposed dosage sensitivity of these genes or could possibly be attributed to the incomplete penetrance of polydactyly in patients with 13q trisomy/tetrasomy. Congenital hemangiomas, seen in this patient, have also been previously reported in six other patients with tetrasomy 13q. The gene *EFNB2*, located at 13q33.3, has been suggested as a candidate gene, overexpression of which could be causative of vascular malformations (Liu et al., 2013). The protein encoded by the *EFNB2* gene is a ligand for receptor tyrosine kinases and is shown to play an essential role in angiogenesis (Wang et al., 2010).

Interestingly, exome sequencing for the suspected vascular malformations in the current patient was negative, and no causative variants were found in vascular disease genes such as *TEK/RASA1/EPHB4*, amongst others. Other proposed candidate genes for vascular malformations in 13q are *COL4A1* and *COL4A2*. *COL4A1* and *COL4A2* are associated with multiple hereditary angiopathy disorders; hence, their potential roles in vascular malformations could be considered but requires further investigation. Minor central nervous system anomalies such as tethered cord and ocular anomalies such as anophthalmia, coloboma, strabismus, and ear anomalies have been reported in multiple patients, including the current patient. However, dosage-sensitive candidate genes possibly responsible for these phenotypes remain to be deciphered. With the continued refinement of next-generation sequencing technologies, isolated smaller copy number gains for different regions of distal 13q, if seen in affected individuals, may provide some clues in future clinical studies.

Chromosome breaks during mitosis, or meiosis can lead to the formation of class I marker chromosomes involving inverted duplications or class II marker chromosomes involving interstitial deletions (Marshall et al., 2008). Given the mosaic nature of the marker chromosome in the proband, it is likely a class I marker chromosome of a postzygotic mitotic origin. This is in contrast to the previous prenatal reports of non-mosaic 13q31 sSMCs (Mascarenhas et al., 2008; Haddad et al., 2012; Stembalska et al., 2015) that most likely involved chromosomal breaks that occurred during meiosis. A possible meiotic origin of sSMCs has also been reported for other chromosomal regions such as 13q32 and 2q33.3, for instance (Rivera et al., 1999; Ma et al., 2015). Additionally, the marker was not detected in parental karyotypes suggesting the *de novo* origin of the marker. Given that the proband was conceived by intrauterine insemination, it raises the question if the occurrence of the marker was related to the use of assisted reproductive technology (ART). The possibility of a link between chromosomal rearrangements and ART has been investigated in multiple large studies. Previous studies suggested an increased risk for chromosomal aberrations with ART (Gjerris et al., 2008). However, these conclusions have not been supported by more recent studies (Kim et al., 2010). Systematic review and meta-analyses of these studies often suggest an ascertainment bias, lack of appropriate control groups, and an increased risk, if found, is not significantly higher than that associated with natural conception in the general population (Berntsen et al., 2021). The more widespread use of ART paired with NIPS in the future should provide more accurate estimates and answers for this often controversial question.

Neocentromeres commonly found in association with these marker chromosomes, while making it a challenge to identify the chromosomal origin of the markers, could also add to the challenges associated with trying to make genotype–phenotype correlations. The phenotypic variability seen in patients with neocentromeric marker chromosomes could likely be an indirect consequence of the local epigenetic changes brought about by the creation of a novel centromere and its effect on the expression of neighboring genes (Levy et al., 2000). Future investigations to understand how neocentromeres could regulate gene expression will open new avenues to identify epigenetic targets for possible treatment in patients. This case report adds to the natural history of prenatally identified neocentric sSMCs. Given the recent advances in prenatal genomic testing, this clinical scenario may be more frequent than one might anticipate in the current fetal medicine.

## Data Availability

The datasets for this article are not publicly available due to concerns regarding participant/patient anonymity. Requests to access the datasets should be directed to the corresponding author.

## References

[B1] Abdelhakim A. H. Dharmadhikari A. V. Ragi S. D. de Carvalho J. R. L. Jr Xu C. L. Thomas A. L. (2020). Compound Heterozygous Inheritance of Two Novel COQ2 Variants Results in Familial Coenzyme Q Deficiency. Orphanet J. Rare Dis. 15 (1), 320. 10.1186/s13023-020-01600-8 33187544 PMC7662744

[B2] Alonso A. Fritz B. Hasson D. Abrusan G. Cheung F. Yoda K. (2007). Co-localization of CENP-C and CENP-H to Discontinuous Domains of CENP-A Chromatin at Human Neocentromeres. Genome Biol. 8 (7), R148. 10.1186/gb-2007-8-7-r148 17651496 PMC2323242

[B3] Alonso A. Mahmood R. Li S. Cheung F. Yoda K. Warburton P. E. (2003). Genomic Microarray Analysis Reveals Distinct Locations for the CENP-A Binding Domains in Three Human Chromosome 13q32 Neocentromeres. Hum. Mol. Genet. 12 (20), 2711–2721. 10.1093/hmg/ddg282 12928482

[B4] American College of Obstetricians and Gynecologists’ Committee on Practice Bulletins—Obstetrics (2020). Committee on Genetics; Society for Maternal-Fetal Medicine. Screening for Fetal Chromosomal Abnormalities: ACOG Practice Bulletin, Number 226. Obstet. Gynecol. 136 (4), e48–e69. 10.1097/AOG.0000000000004084 32804883

[B5] Arsham M. S. Barch M. J. Lawce H. J. (2017). The AGT Cytogenetics Laboratory Manual. Hoboken, NJ: John Wiley & Sons, Inc. 4th ed.

[B6] Barwell J. Mazzaschi R. Bint S. Ogilvie C. M. Elmslie F. (2004). A New Neocentromere Locus on Chromosome 13 Resulting in Mosaic Tetrasomy for Distal 13q and an Asymmetric Phenotype. Am. J. Med. Genet. 130A (3), 295–298. 10.1002/ajmg.a.30208 15378552

[B7] Berntsen S. Laivuori H. la Cour Freiesleben N. Loft A. Söderström-Anttila V. B Oldereid N. (2021). A Systematic Review and Meta-Analysis on the Association between ICSI and Chromosome Abnormalities. Hum. Reprod. Update 27 (5), 801–847. 10.1093/humupd/dmab005 33956940

[B8] Chitty L. S. (2021). Non‐invasive Prenatal Testing 10 Years on. Prenat. Diagn. 41 (10), 1187–1189. 10.1002/pd.6032 34418119

[B9] Chueh A. C. Wong L. H. Wong N. Choo K. H. A. (2005). Variable and Hierarchical Size Distribution of L1-Retroelement-Enriched CENP-A Clusters within a Functional Human Neocentromere. Hum. Mol. Genet. 14 (1), 85–93. 10.1093/hmg/ddi008 15537667

[B10] Dhar S. U. Robbins-Furman P. Levy M. L. Patel A. Scaglia F. (2009). Tetrasomy 13q Mosaicism Associated with Phylloid Hypomelanosis and Precocious Puberty. Am. J. Med. Genet. 149A (5), 993–996. 10.1002/ajmg.a.32758 19334087 PMC3587162

[B11] Ganapathi M. Nahum O. Levy B. (2019). Prenatal Diagnosis Using Chromosomal SNP Microarrays. Methods Mol. Biol. 1885, 187–205. 10.1007/978-1-4939-8889-1_13 30506199

[B12] Gjerris A. C. Loft A. Pinborg A. Christiansen M. Tabor A. (2008). Prenatal Testing Among Women Pregnant after Assisted Reproductive Techniques in Denmark 1995-2000: a National Cohort Study. Hum. Reprod. 23 (7), 1545–1552. 10.1093/humrep/den103 18385126

[B13] Haddad V. Aboura A. Tosca L. Guediche N. Mas A.-E. L'Herminé A. C. (2012). Tetrasomy 13q31.1qter Due to an Inverted Duplicated Neocentric Marker Chromosome in a Fetus with Multiple Malformations. Am. J. Med. Genet. 158A (4), 894–900. 10.1002/ajmg.a.35258 22419357

[B14] Jobanputra V. Burke A. Kwame A.-Y. Shanmugham A. Shirazi M. Brown S. (2012). Duplication of the ZIC2 Gene Is Not Associated with Holoprosencephaly. Am. J. Med. Genet. 158A (1), 103–108. 10.1002/ajmg.a.34375 22105922 PMC3715301

[B15] Kim J. W. Lee W. S. Yoon T. K. Seok H. H. Cho J. H. Kim Y. S. (2010). Chromosomal Abnormalities in Spontaneous Abortion after Assisted Reproductive Treatment. BMC Med. Genet. 11, 153. 10.1186/1471-2350-11-153 21044350 PMC2991301

[B16] Krygier M. Lipska-Zietkiewicz B. S. Koczkowska M. Wierzba J. Limon J. (2014). Mild Phenotype of a Large Partial 13q Trisomy. Clin. Dysmorphol. 23 (4), 155–157. 10.1097/MCD.0000000000000052 25144153

[B17] Levy B. Papenhausen P. Tepperberg J. Dunn T. Fallet S. Magid M. (2000). Prenatal Molecular Cytogenetic Diagnosis of Partial Tetrasomy 10p Due to Neocentromere Formation in an Inversion Duplication Analphoid Marker Chromosome. Cytogenet Cell. Genet. 91 (1-4), 165–170. 10.1159/000056839 11173851

[B18] Li S. Malafiej P. Levy B. Mahmood R. Field M. Hughes T. (2002). Chromosome 13q Neocentromeres: Molecular Cytogenetic Characterization of Three Additional Cases and Clinical Spectrum. Am. J. Med. Genet. 110 (3), 258–267. 10.1002/ajmg.10454 12116235

[B19] Liehr T. (2021). Non-invasive Prenatal Testing, what Patients Do Not Learn, May Be Due to Lack of Specialist Genetic Training by Gynecologists and Obstetricians? Front. Genet. 12, 682980. 10.3389/fgene.2021.682980 34220958 PMC8248176

[B20] Liu J. Jethva R. Del Vecchio M. T. Hauptman J. E. Pascasio J. M. de Chadarévian J.-P. (2013). Tetrasomy 13q32.2qter Due to an Apparent Inverted Duplicated Neocentric Marker Chromosome in an Infant with Hemangiomas, Failure to Thrive, Laryngomalacia, and Tethered Cord. Birth Defects Res. Part A Clin. Mol. Teratol. 97 (12), 812–815. 10.1002/bdra.23197 24222317

[B21] Lo A. W. I. Craig J. M. Saffery R. Kalitsis P. Irvine D. V. Earle E. (2001). A 330 Kb CENP-A Binding Domain and Altered Replication Timing at a Human Neocentromere. EMBO J. 20 (8), 2087–2096. 10.1093/emboj/20.8.2087 11296241 PMC125239

[B22] Lo A. W. I. Magliano D. J. Sibson M. C. Kalitsis P. Craig J. M. Choo K. H. A. (2001). A Novel Chromatin Immunoprecipitation and Array (CIA) Analysis Identifies a 460-kb CENP-A-Binding Neocentromere DNA. Genome Res. 11 (3), 448–457. 10.1101/gr.167601 11230169 PMC311059

[B23] Luo Y. Lin J. Sun Y. Qian Y. Wang L. Chen M. (2020). Non-invasive Prenatal Screening for Emanuel Syndrome. Mol. Cytogenet 13, 9. 10.1186/s13039-020-0476-7 32158503 PMC7057502

[B24] Ma R. Peng Y. Zhang Y. Xia Y. Tang G. Chang J. (2015). Partial Trisomy 2q33.3-q37.3 in a Patient with an Inverted Duplicated Neocentric Marker Chromosome. Mol. Cytogenet 8, 10. 10.1186/s13039-015-0111-1 25774219 PMC4359772

[B25] Marshall O. J. Chueh A. C. Wong L. H. Choo K. H. A. (2008). Neocentromeres: New Insights into Centromere Structure, Disease Development, and Karyotype Evolution. Am. J. Hum. Genet. 82 (2), 261–282. 10.1016/j.ajhg.2007.11.009 18252209 PMC2427194

[B26] Mascarenhas A. Matoso E. Saraiva J. Tönnies H. Gerlach A. Julião M. J. (2008). First Prenatally Detected Small Supernumerary Neocentromeric Derivative Chromosome 13 Resulting in a Non-mosaic Partial Tetrasomy 13q. Cytogenet Genome Res. 121 (3-4), 293–297. 10.1159/000138901 18758175

[B27] Murmann A. E. Conrad D. F. Mashek H. Curtis C. A. Nicolae R. I. Ober C. (2009). Inverted Duplications on Acentric Markers: Mechanism of Formation. Hum. Mol. Genet. 18 (12), 2241–2256. 10.1093/hmg/ddp160 19336476 PMC2685760

[B28] Myers J. N. Jr Davis L. Sheehan D. Kulharya A. S. (2015). Mosaic Tetrasomy 13q and Phylloid Hypomelanosis: a Case Report and Review of the Literature. Pediatr. Dermatol 32 (2), 263–266. 10.1111/pde.12375 24920397

[B29] Naughton C. Gilbert N. (2020). Centromere Chromatin Structure - Lessons from Neocentromeres. Exp. Cell. Res. 389 (2), 111899. 10.1016/j.yexcr.2020.111899 32044308

[B30] Rivera H. Vasquez A. I. Garcia-Cruz D. Crolla J. A. (1999). Neocentromere at 13q32 in One of Two Stable Markers Derived from a 13q21 Break. Am. J. Med. Genet. 85 (4), 385–388. 10.1002/(sici)1096-8628(19990806)85:4<385::aid-ajmg15>3.0.co;2-p 10398265

[B31] Stembalska A. Jagielska G. Laczmanska I. Szmida E. Jarczynska A. Gil J. (2015). Hexasomy 13q31.3q34 Due to Two Marker Chromosomes with Inverted Duplication in a Fetus with Increased Nuchal Translucency. Birth Defects Res. Part A Clin. Mol. Teratol. 103 (4), 255–259. 10.1002/bdra.23344 25852029

[B32] Tharapel S. A. Lewandowski R. C. Tharapel A. T. Wilroy R. S. Jr (1986). Phenotype-karyotype Correlation in Patients Trisomic for Various Segments of Chromosome 13. J. Med. Genet. 23 (4), 310–315. 10.1136/jmg.23.4.310 3746829 PMC1049695

[B33] Tohma T. Ohashi H. Hasegawa T. Nagai T. Fukushima Y. Naritomi K. (1998). Two Cases of Mosaic Partial Tetrasomy 13q Associated with an Acentric Marker Chromosome. Am. J. Hum. Genet. 63 (Suppl. l), A862.

[B34] van der Zwaag P. A. Dijkhuizen T. Gerssen-Schoorl K. B. J. Colijn A. W. Broens P. M. A. Flapper B. C. T. (2010). An Interstitial Duplication of Chromosome 13q31.3q32.1 Further Delineates the Critical Region for Postaxial Polydactyly Type A2. Eur. J. Med. Genet. 53 (1), 45–49. 10.1016/j.ejmg.2009.11.003 19941983

[B35] Veugelers M. De Cat B. Ceulemans H. Bruystens A.-M. Coomans C. Dürr J. (1999). Glypican-6, a New Member of the Glypican Family of Cell Surface Heparan Sulfate Proteoglycans. J. Biol. Chem. 274 (38), 26968–26977. 10.1074/jbc.274.38.26968 10480909

[B36] Voullaire L. E. Slater H. R. Petrovic V. Choo K. H. (1993). A Functional Marker Centromere with No Detectable Alpha-Satellite, Satellite III, or CENP-B Protein: Activation of a Latent Centromere? Am. J. Hum. Genet. 52 (6), 1153–1163. 7684888 PMC1682274

[B37] Wang Y. Nakayama M. Pitulescu M. E. Schmidt T. S. Bochenek M. L. Sakakibara A. (2010). Ephrin-B2 Controls VEGF-Induced Angiogenesis and Lymphangiogenesis. Nature 465 (7297), 483–486. 10.1038/nature09002 20445537

[B38] Warburton P. E. Dolled M. Mahmood R. Alonso A. Li S. Naritomi K. (2000). Molecular Cytogenetic Analysis of Eight Inversion Duplications of Human Chromosome 13q that Each Contain a Neocentromere. Am. J. Hum. Genet. 66 (6), 1794–1806. 10.1086/302924 10777715 PMC1378043

[B39] Yu S. Fiedler S. D. Brawner S. J. Joyce J. M. Zhou X. G. Liu H. Y. (2012). Characterizing Small Supernumerary Marker Chromosomes with Combination of Multiple Techniques. Cytogenet Genome Res. 136 (1), 6–14. 10.1159/000334271 22123409

