# Supplementary material for: Case Report: Prenatal Identification of a De Novo Mosaic Neocentric Marker Resulting in 13q31.1→qter Tetrasomy in a Mildly Affected Girl
Source: Front Genet. 2022 Jul 19;13:906077. doi: 10.3389/fgene.2022.906077 (PMC9343796; doi:10.3389/fgene.2022.906077)
Supplement: Supplementary file 1 [file Table1.DOCX]

**Supplementary Table 1:** **Comparison of clinical phenotypes observed in the current proband with previously reported sSMCs cases with breakpoints in 13q31.**

|  | **Current Study** | **Tohma et al. 1998 [27]** | **Li et al. 2002 [12]** | **Barwell et al. 2004 [19]** | **Yu et al. 2011 [16]** | **Mascarenhas et al. 2008 [13]** | **Haddad et al. 2012 [14]** | **Stembalska et al. 2015 [15]** |
| --- | --- | --- | --- | --- | --- | --- | --- | --- |
| **Head &Neck** | Bulbous nasal tip, arched palate, slight neck skin redundancy | Thick eyebrows, hypertelorism | Bitemporal narrowing, hypotelorism, tented upper lip | Long smooth philtrum, Right torticollis, head circumference (98^th^ percentile) | Cleft palate | Short nose and neck, hypotelorism, short palpebral fissures | V-shaped metopic suture, short, flattened nose, short buccal frenulum, cystic cervical hygroma, head circumference (10^th^ percentile) | Increased nuchal fold, wide neck, dysmorphic facial features |
| **Eyes** | Left microphthalmia, oculomotor apraxia, strabismus, congenital anomaly of the right optic nerve | Strabismus | Strabismus | Bilateral epicanthic folds | **-** | **-** | Deep-set eyes | **-** |
| **Ears** | Slight posterior rotation of the left ear, uplifted ear lobes, | Deformed lobes | - | Unilateral hearing loss, large fleshly ear lobe | **-** | Low set ears | Small and low-set ears | **-** |
| **Teeth** | - | Three extra teeth in lower jaw | Extra low incisor | Complete extra set of teeth | **-** | **-** | **-** | **-** |
| **Growth** | - | - | **-** | Arm hemihypertrophy | **-** | **-** | **-** | Head and body disproportion |
| **Cardiovascular** | - | Patent ductus arteriosus | **-** | **-** | **-** | **-** | **-** | **-** |
| **Respiratory** | Neonatal respiratory distress | Bronchial anomalies | **-** | **-** | **-** | **-** | **-** | **-** |
| **Chest** | **-** | Diaphragmatic hernia | **-** | **-** | **-** | **-** | **-** | Left-sided diaphragmatic hernia |
| **Abdomen** | **-** | Intestinal malrotation | **-** | **-** | **-** | **-** | Malrotation of the gut | Incorrect positing of anus, |
| **Genitourinary** | **-** | Hypospadias, hydronephrosis | **-** | **-** | **-** | Enlarged and hyperechogenic kidneys | Hyper echogenic and large kidneys, micropenis with hypospadias | Ambiguous genitalia, hydronephrosis |
| **Skeletal** | Bilateral fifth finger and toe clinodactyly | Scoliosis | Clinodactyly | **-** | **-** | Club left foot | Postaxial polydactyl of the right hand and left foot with short fingers, short, long bones | Shortening of long bones |
| **Skin, nails, &Hair** | Hemangiomas (red pigmentation over her face, neck, buttocks, back, and genitalia) | - | Nevus flammeus on nasal bridge | **-** | **-** | **-** | **-** | **-** |
| **Muscle, soft tissues** | Hypotonia | - | **-** | **-** | **-** | **-** | **-** | **-** |
| **Neurologic** | Gross motor delay, speech delay, tethered spinal cord requiring laminectomy, thinning of the corpus callosum | Learning difficulties, seizures | Mild developmental delays | Mild motor developmental delay, learning difficulties, seizures | Learning difficulties, cleft palate, seizures | Large cisterna magna, ventriculomegaly | Mega cisterna, cerebellar hypoplasia | **-** |
| **Prenatal manifestations** | Oligohydramnios | - | - | - | - | Oligohydramnios, | Intra-uterine growth restriction | **-** |
| **Immunology** | - | - | - | - | - | Thymic hypoplasia | **-** | **-** |
| **Age at report** | 21 months | 4 years | 4 years | 8 years | 15 years | Pregnancy terminated after 23 weeks estimated gestational age | Pregnancy terminated at 17 weeks estimated gestational age | Pregnancy terminated at 18 weeks estimated gestational age |
